# Deep reefs are not refugium for shallow‐water fish communities in the southwestern Atlantic

**DOI:** 10.1002/ece3.7336

**Published:** 2021-03-18

**Authors:** Aline P. M. Medeiros, Beatrice P. Ferreira, Fredy Alvarado, Ricardo Betancur‐R, Marcelo O. Soares, Bráulio A. Santos

**Affiliations:** ^1^ Programa de Pós‐Graduação em Ciências Biológicas Universidade Federal da Paraíba João Pessoa PB Brazil; ^2^ Departamento de Oceanografia Centro de Tecnologia Universidade Federal de Pernambuco Recife Brazil; ^3^ Departamento de Agricultura Centro de Ciências Humanas Sociais e Agrárias Universidade Federal da Paraíba Bananeiras PB Brazil; ^4^ Department of Biology The University of Oklahoma Norman OK USA; ^5^ Department of Vertebrate Zoology National Museum of Natural History Smithsonian Institution Washington DC USA; ^6^ Instituto de Ciências do Mar‐LABOMAR Universidade Federal do Ceará Meireles Brazil; ^7^ Institut de Ciència i Tecnologia Ambientals (ICTA) Universitat Autònoma de Barcelona (UAB) Barcelona Spain; ^8^ Dipartimento di Scienze e Tecnologie Biologiche e Ambientali (DISTEBA) Università del Salento Lecce Italy; ^9^ Departamento de Sistemática e Ecologia Centro de Ciências Exatas e da Natureza Universidade Federal da Paraíba João Pessoa PB Brazil

**Keywords:** coral reefs, depth, fish, mesophotic coral ecosystems

## Abstract

1. The deep reef refugia hypothesis (DRRH) predicts that deep reef ecosystems may act as refugium for the biota of disturbed shallow waters. Because deep reefs are among the most understudied habitats on Earth, formal tests of the DRRH remain scarce. If the DRRH is valid at the community level, the diversity of species, functions, and lineages of fish communities of shallow reefs should be encapsulated in deep reefs.
2. We tested the DRRH by assessing the taxonomic, functional, and phylogenetic diversity of 22 Brazilian fish communities between 2 and 62 m depth. We partitioned the gamma diversity of shallow (<30 m) and deep reefs (>30 m) into independent alpha and beta components, accounted for species’ abundance, and assessed whether beta patterns were mostly driven by spatial turnover or nestedness.
3. We recorded 3,821 fishes belonging to 85 species and 36 families. Contrary to DRRH expectations, only 48% of the species occurred in both shallow and deep reefs. Alpha diversity of rare species was higher in deep reefs as expected, but alpha diversity of typical and dominant species did not vary with depth. Alpha functional diversity was higher in deep reefs only for rare and typical species, but not for dominant species. Alpha phylogenetic diversity was consistently higher in deep reefs, supporting DRRH expectations.
4. Profiles of taxonomic, functional, and phylogenetic beta diversity indicated that deep reefs were not more heterogeneous than shallow reefs, contradicting expectations of biotic homogenization near sea surface. Furthermore, pairwise beta‐diversity analyses revealed that the patterns were mostly driven by spatial turnover rather than nestedness at any depth.
5. *Conclusions*. Although some results support the DRRH, most indicate that the shallow‐water reef fish diversity is not fully encapsulated in deep reefs. Every reef contributes significantly to the regional diversity and must be managed and protected accordingly.

## Graphical abstract

The taxonomic, functional, and phylogenetic diversity of shallow‐water reef fish communities is not fully encapsulated in deep reefs. Every reef contributes significantly to the regional fish diversity. Deep and shallow reefs complement each other and must be managed and protected accordingly.

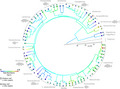

## 1 INTRODUCTION

As shallow‐water coral reef ecosystems are gradually degraded by human activities, identifying areas where biodiversity can be maintained has become a conservation priority worldwide (Morais et al., 2018; Soares et al., 2020). These areas comprise marginal reefs such as turbid‐zone and high‐temperature areas, macrotidal reefs, tide pools, volcanic CO_2_ vents, *ojos* (low pH springs), areas with submarine groundwater discharge, mangrove systems, upwelling areas, temperate mesophotic ecosystems, mesophotic coral ecosystems, and cold‐water coral ecosystems (Camp et al., 2018; Enochs et al., 2020; Soares et al., 2021). Growing attention has been paid to the mesophotic coral ecosystems (deep reefs hereafter), which are usually characterized by the presence of light‐dependent corals and other habitat‐forming benthic organisms (i.e., octocorals, antipatharians, macroalgae, and sponges) that are typically found at depths ranging from 30 to 150 m in tropical and subtropical regions (Asher et al., 2017; Hinderstein et al., 2010; Pinheiro et al., 2019; Pyle & Copus, 2019; Rosa et al., 2016).

The deep reefs are closely linked to shallow reef areas, usually forming a contiguous or semi‐contiguous belt of habitats along a depth gradient (Lindfield et al., 2016). However, unlike the shallow reefs (<30 m depth), the deep reefs are presumably less exposed to ocean warming and other human pressures such as coastal pollution, overfishing, and nonregulated tourism (Hoegh‐Guldberg & Bruno, 2010; Mies et al., 2020; Mora et al., 2011; Soares et al., 2020), leading researchers to postulate that deep reefs could act as depth refuge, refugium, or resilience area for reef biota in face of a massive loss of shallow reefs ("deep reef refugia hypothesis" sensu Bongaerts et al., 2010; see also Glynn, 1996; Kahng et al., 2010; Loya et al., 2016). By *depth refuge,* we mean a depth range that provides short‐term buffering or shelter for one or multiple species or a biological community against a particular disturbance episode; *depth refugium*, a depth range that provides a long‐term buffering or shelter for one or multiple species or a biological community against a particular or multiple disturbance types; and *resilience area*, a depth range that harbors one or multiple species or a biological community that is/are resilient over the long term to a particular or multiple disturbance types (sensu Bongaerts & Smith, 2019).

The potential of deep reefs as depth refuges, refugia, and resilience areas has been assessed at different regions around the world (Rocha et al., 2018), such as the Great Barrier Reef (Jankowski et al., 2015), Micronesia (Coleman et al., 2018), Mariana Islands (Lindfield et al., 2016), and the Coral Triangle (Andradi‐Brown et al., 2019). Nonetheless, to date there is no consensus on the role of deep reefs to fully encapsulate the diversity of shallow‐water communities (Bongaerts et al., 2017; Morais & Santos, 2018; Pereira et al., 2018; Semmler et al., 2017). The biological level of the studies (e.g., population or community), the ecological group (e.g., invertebrate or vertebrate, mobile or sessile), the level of structural connectivity (contiguous or separated) between shallow and deep reefs, the different diversity metrics selected by researchers (e.g., species richness per se, presence/absence indices), and weak theoretical foundations are among the major reasons that have impaired the consensus.

The metacommunity theory combined with reliable metrics of community diversity provides a useful framework to test the DRRH. If the hypothesis is valid at the community level, the metacommunity—the entire gradient of deep and shallow local communities—must be mainly structured by mass effects (sensu Leibold et al., 2004). Under this scenario, local environmental conditions are less important than dispersal capacity and species may colonize any site along the depth gradient, becoming more abundant in sites where conditions are more suitable. High reproduction rates in suitable sites allow populations to export individuals to unsuitable sites and protect smaller populations from local extinction (Leibold et al., 2004). When these rescue effects are scaled up to communities, compositional similarity between suitable and unsuitable sites tends to increase, with local communities of unsuitable sites being nested within suitable sites. From the DRRH perspective, the unsuitable sites are represented by the shallow disturbed reefs, which home only a small number of stress‐tolerant species that tend to homogenize the reefs and impoverish the shallow region, resulting in low alpha, beta, and gamma diversity. Conversely, the deep conserved reefs represent the suitable sites, where a greater number of species coexist locally, replace each other from one reef to another, and comprise a species‐rich region, resulting in high alpha, beta, and gamma diversity. This rationale may be applied not only for the diversity of species as commonly observed in the literature, but also for functions and lineages.

Here, we tested the DRRH for reef fishes using a community‐level approach able to partition the taxonomic, functional, and phylogenetic gamma diversity into independent alpha and beta components (Jost, 2007). We classified the species into six complementary functional traits to examine the role of depth in safeguarding functions of fish communities. We also estimated a phylogenetic tree using maximum‐likelihood and backbone constraint analyses to calculate the phylogenetic diversity. We sampled 22 reef fish communities in Northeast Brazil to assess six predictions derived from the DRRH (see Morais & Santos, 2018, for a similar study with corals): (a) Depth‐generalist fish species should dominate the metacommunity because if species are exclusive to shallow or deep areas they could not be rescued from eventual local extinction; (b) deep reefs should contain greater gamma diversity than shallow reefs to be able to export species to and replenish shallow reefs, provided that species composition of the shallow areas is nested within the deep areas; (c) alpha diversity should be greater in deep reefs due to reduced anthropogenic pressure in deeper areas; (d) regional beta diversity should be smaller among shallow reefs than among deep reefs owing to increased disturbance and biotic homogenization driven by the proliferation of stress‐tolerant species near sea surface; (e) pairwise beta patterns of shallow reefs should be mostly driven by nestedness than spatial turnover, while nested effects should become weaker between deep reefs; and (f) functional diversity of shallow communities should be a subset of the functional diversity of deep communities, with exclusive functional traits observed only in deep reefs.

## 2 MATERIALS AND METHODS

### 2.1 Study area

**FIGURE 1:**
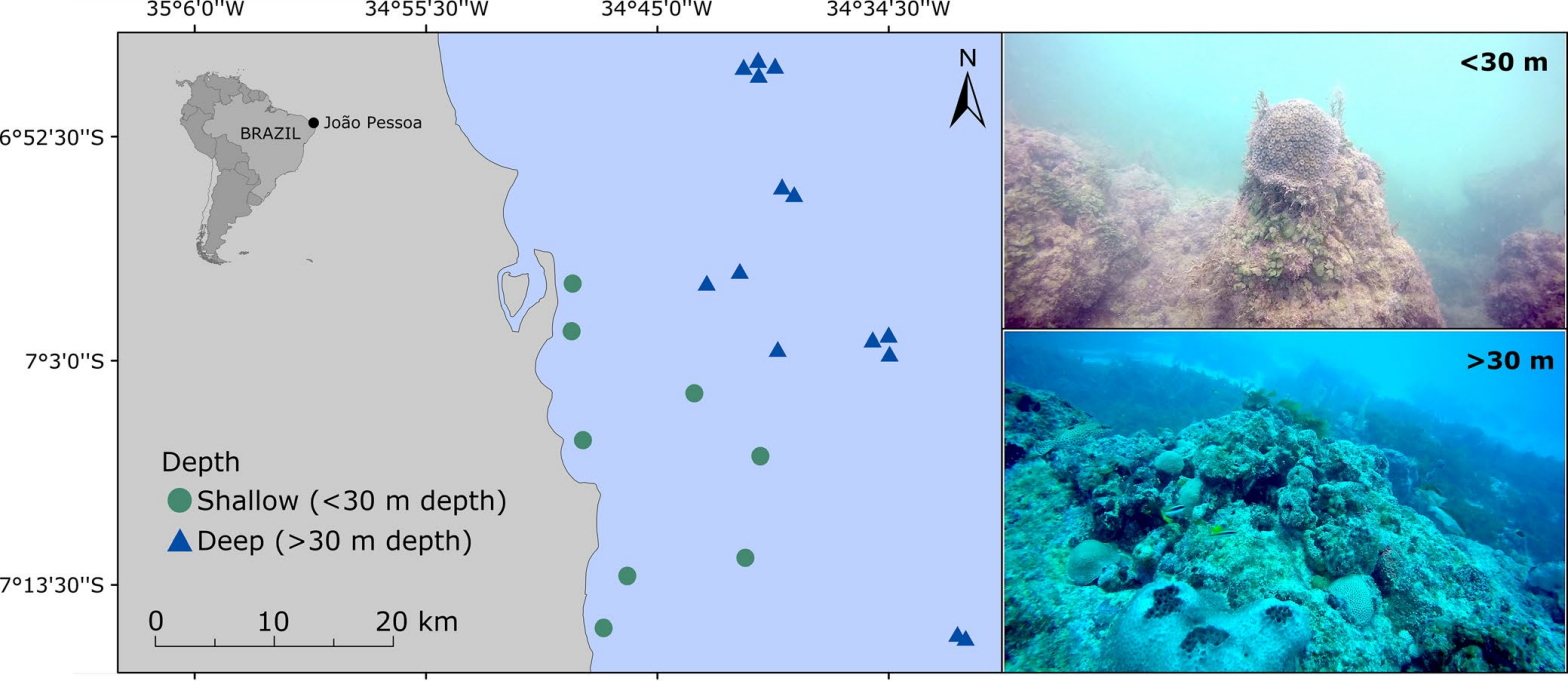
Study region in the coast of Paraiba, southwestern Atlantic, showing an example of shallow (<30 m depth; green circles) and deep reefs (>30 m depth; blue triangles)

We carried out the study in southwestern Atlantic reef ecosystems located along the Northeastern Brazilian subprovince (Pinheiro et al., 2018; between 7°0′023S 34°50′0″W and 7°15′0″S 34°30′0″W; Figure 1). The region is under influence of the South Equatorial Current and has the water temperature ranging from 23 to 30°C, with a thermocline found at about 50 m depth where water temperature declines to 23°C and visibility increases from 20 to 50 m (Feitoza et al., 2005; Maida & Ferreira, 1997; Rocha, 2003). This area is known for having rock‐based reefs of various shapes and dimensions forming lines parallel to the coastline, with a sharp decline of the seafloor at near 70 m depth (Feitoza et al., 2005; Leão & Dominguez, 2000). The reefs are covered by extensive growths of benthic organisms, especially calcareous algae, macroalgae, and macrobenthos (i.e., Zoanthidae and sponges) (Honório et al., 2010); coral cover varies from 0.3% to 20% (Morais & Santos, 2018). Reef fish composition of shallow areas is well‐studied (Feitoza et al., 2002; Honório et al., 2010; Osório et al., 2006; Ramos, 1994; Rocha et al., 2000; Rocha et al., 1998; Rosa et al., 1997; Silva et al., 2014; Souza et al., 2007), while fishes of deep reefs are poorly known (but see Feitoza et al., 2005). Common disturbances of shallow reefs include mass tourism, pollution, and overfishing (Leão et al., 2016). For instance, Medeiros et al. (2007) documented the effects of tourism in the study region, including community homogenization, changes in the trophic structure, and the dominance of stress‐tolerant species. Disturbance of deep reefs is scantly documented, but fishermen report overfishing.

### 2.2 Data survey

We surveyed 22 coral reefs ranging from 2 to 62 m maximum depth. Reefs were categorized into shallow (<30 m depth; *n* = 8 reefs) and deep (>30 m depth; *n* = 14 reefs). To sample fish communities at each reef, we performed SCUBA dives using high‐resolution video records (GoPro Hero 4) and following the browsing trajectory technique (Mallet & Pelletier, 2014; Mallet et al., 2016). Video recording was performed with slow movement at approximately 1 m above the bottom. The goal was to comprise all the reef extent. Assuming that each reef has different shapes and features (e.g., number of crevices), we aimed to record all the extent of each reef. This approach allowed to focus in different available habitats (i.e., bottom reef, interface, and water column) to record the entire coral reef fish community (Mallet & Pelletier, 2014; Meirelles et al., 2015; Pelletier et al., 2011). This way, each reef had different recording times and trajectories, but because samples were standardized by completeness rather than size as recommend by Chao and Jost (2012) (see Section 2.3), our statistical analyses were reliable and reproducible (Cardoso et al., 2020). Videos were later analyzed to identify fish species and estimate their abundance. Identification was conducted based on our own field experience and local field guides. Species abundance was measured as the maximum number conspecifics seen simultaneously in the same frame (Barley et al., 2017; Lindfield et al., 2016).

We classified the species into six functional traits that defined the functional profile of fish communities in terms of species’ behavior, habitat use, body size, and trophic niche: (a) water column position (benthic; pelagic; benthopelagic); (b) habitat use (generalist; intermediate generalist; specialist); (c) body size categories based on the total length recorded in literature (0–7 cm; 7.1–15 cm; 15.1–30 cm; 30.1–50 cm; 50.1–80 cm; >80 cm); (d) mobility (high mobility; roving; sedentary); (e) trophic categories (herbivore; macrocarnivore; macroinvertivore; small invertivore; omnivore; planktivore); and (f) spawning mode (Balistidae type; brooding; demersal eggs; pelagic eggs; ovoviviparous). We chose these functional traits based on the literature available and on the complementary functions they represent, including habitat use, food acquisition, mobility, nutrient budget, and reproduction strategies (Villéger et al., 2017). Most functional trait data were obtained from Pinheiro et al. (2018, available at https://swatlanticreeffishes.wordpress.com) and complemented with our own field records (e.g., water column).

**FIGURE 2:**
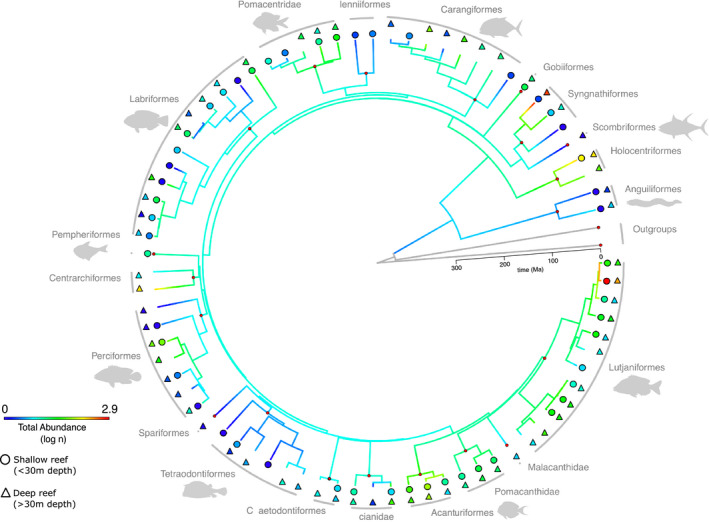
Time‐calibrated phylogeny containing 77 of the 85 species recorded in the present study. Internal red circles represent taxonomic annotation (e.g., order) clades of Teleostei and the two outgroups. For visualization purposes, branch colors indicate ancestral abundance reconstruction for the Teleostei species (see Methods for details). Circles and triangles represent the abundance of species in shallow and deep areas, respectively; symbol color indicates species abundance

To calculate the phylogenetic diversity, we estimated a phylogenetic tree for 77 Teleostei fish species of the 85 recorded (Figure 2). We retrieved 704 *cytochrome oxidase subunit I* (COI) and 214 *cytochrome b* (Cytb) sequences from NCBI for the species and aligned the sequences using MUSCLE v3.8.425 (Edgar, 2004) as implemented in Geneious Prime 2019.1.1 (https://www.geneious.com). The next step was to assemble gene trees at family or order level, depending on the number of sequences available, which allowed us to identify misplaced sequences. This quality control step was conducted to avoid misidentifications or any discrepancies in the sequence selection. The phylogenetic tree was then estimated using maximum‐likelihood (ML) and backbone constraint analyses. The backbone tree used was based on a multilocus phylogenetic analysis of ray‐finned fishes dated with multiple fossil calibration points containing 1,661 species (Betancur‐R et al., 2017). Of the 77 species with COI and/or Cytb data, 31 were previously placed in the backbone tree (accession numbers are given in Table S1). Our aim was to obtain phylogenetic placement for the remaining, previously unexamined 46 species. We conducted constraint ML searches in RAxML v8.1.20 using by‐codon partitions and 10 independent iterations (Stamatakis, 2006; Stamatakis et al., 2008), and time‐calibrated the resulting ML tree in TreePL v1.0 (Smith & O’Meara, 2012). The TreePL analysis was based on secondary calibrations extracted from the reference backbone tree via “congruification” (Eastman et al., 2013), a function (“congruify”) implemented in the R package *geiger* (Harmon et al., 2008). We then pruned the resulting tree to retain the 77 target species using the R package *phytools* (Revell, 2012). Eight species recorded in the study, which accounted for 0.8% of the total abundance registered, were not represented in the tree, including three elasmobranchs and five Teleostei. Although the recent debates regarding the status of Epinephelinae as a separated family from Serranidae, we maintained the nomenclature following the suggestions indicated in Betancur‐R et al. (2017), which recognizes the monophyly of Serranidae. The final time‐calibrated tree was used to measure the phylogenetic diversity with the R package *hillR* (Chao et al., 2014).

### 2.3 Data analysis

To compare fish species diversity across the depth gradient, we standardized the 22 sampled reefs by completeness rather than size, as recommended by Chao and Jost (2012). Accordingly, we estimated the sample coverage in each reef and for each depth category (shallow and deep) as follows:

$${\hat{C}}_{n}=1‐\frac{f_{1}}{n}\left[\frac{\left(n‐1\right)f_{1}}{\left(n‐1\right)f_{1}+2f_{2}}\right].$$

where *f_1_* and *f_2_* correspond to singletons and doubletons in the sample, respectively, and *n* is the original sample size (fish abundance in each reef). Sample coverage varied from 69% to 99% (mean 87%) when each reef was analyzed separately and was nearly complete when reefs were collapsed in shallow and deep regions (98% and 99%, respectively) (Table 1). Calculations were performed with the R package *iNext* (Hsieh et al., 2016). We also performed complementary species composition analyses to estimate potential spatial autocorrelation between the reefs. Mantel test indicated that species composition was not correlated with geographic distance (Appendix S1).

**TABLE 1:** Information on depth, sampling effort, and sampling coverage of each reef studied in Northeast Brazil

Reef code	Depth (m)	Depth category	Sampling effort (min)	Sample coverage (%)	Sample coverage by depth category
1	2.4	Shallow	31'26''	85	Shallow 98%
2	2.6	Shallow	23'48''	83	
3	7.7	Shallow	40'04''	89	
4	9.1	Shallow	44'30''	89	
5	9.3	Shallow	33'53''	98	
14	18	Shallow	19'41''	72	
6	20.1	Shallow	28'09''	86	
7	24.5	Shallow	24'19''	80	
8	30	Deep	23'03''	75	Deep 99.7%
9	30	Deep	26'16''	96	
10	30	Deep	31'50''	99	
11	33	Deep	37'43''	96	
12	33	Deep	34'28''	98	
13	33	Deep	28'24''	93	
15	34	Deep	70'19''	89	
16	35	Deep	23'30''	69	
17	35	Deep	20'30''	93	
18	40	Deep	26'02''	95	
19	44.7	Deep	105'01''	96	
20	48	Deep	35'26''	95	
21	54.4	Deep	66'05	89	
22	62.3	Deep	53'09''	97	

Gamma and alpha diversity of species, functions, and lineages was estimated based on the effective number of species, the total functional distance between species of the community, and the effective number of phylogenetic entities, respectively (so‐called Hill numbers *^q^D*; see also Araújo et al., 2020, and Cardoso et al., 2020). Hill numbers are a family of diversity measures developed by Hill (1973) that quantify diversity in units of equivalent numbers of equally abundant species (Gotelli & Chao, 2013). The index allows to exponentially weight species abundance by a *q* factor, and unlike traditional diversity metrics, it satisfies the mathematical replication principle (Chao et al., 2014; Jost, 2010). For *S* species, gamma and alpha diversity of order *q* is defined as follows:

$$D^{q}={\left(\sum_{i=1}^{S}p_{i}^{q}\right)}^{1/\left(1‐q\right)}$$

where *S* is the number of fish species in a reef, *p_i_* is the abundance of the *i*th species, and *q* is the order that controls the sensitivity to species abundance. When *q* = 0 (^0^ *D*), all abundances return to 1 and the index is equivalent to species richness (also known as the diversity of rare species); when *q* = 1 (^1^ *D*), the index maintains the relative abundance of each species and describes the diversity of typical species; and when *q* = 2 (^2^ *D*), the abundances are squared, giving more weight to the more abundant species and indicating the diversity of dominant species (Chao et al., 2014).

When species are examined by a set of traits that describe ecological functions, species differences can be measured by the dissimilarity or distances between their trait profile (Chao et al., 2014). To construct such functional profiles from qualitative traits and estimate functional diversity, we used Gower distance matrix (Chao et al., 2014; Chiu & Chao, 2014). To calculate alpha and gamma functional diversity, we used the total functional distance between species of the community as follows:

$$\text{FD}{\left(Q\right)}^{q}={\left[\sum_{i=1}^{S}\sum_{j=1}^{S}d_{\mathrm{ij}}{\left(\frac{pi\;pj}{Q}\right)}^{q}\right]}^{1/\left(1‐q\right)}$$

where *d_ij_* denotes the functional distance between the *i*th and *j*th species, *p_i_p_j_/Q* denotes Rao's quadratic entropy, and *q* is the order that controls the sensitivity to species abundance. Besides estimating functional diversity based on Hill numbers, we also measured the community‐weighted trait means (CWMs hereafter) to identify which traits were responsible for the functional differences between shallow and deep reefs. CWM is defined as the mean of values present in the community weighted by the abundance of a taxa bearing each attribute value (Lavorel et al., 2008).

Alpha and gamma phylogenetic diversity was also measured under a species‐neutral approach that satisfies the replication principle using the Hill number framework (Chao et al., 2014). Phylogenetic diversity was estimated as follows:

$$\text{PD}{\left(T\right)}^{q}={\left\{\sum_{i\in B_{T}}L_{i}{\left(\frac{a_{i}}{T}\right)}^{q}\right\}}^{1/\left(1‐q\right)}$$

where *L_i_* is the length (or duration) of branch *i* in the set *B_T_*, *a_i_* corresponds to the total abundance descended from branch *i*, and *q* is the order that controls the sensitivity to species abundance. The metric expresses the effective number of maximally distinct lineages over a time interval (Chao et al., 2010).

We applied two approaches to examine beta‐diversity patterns: multiplicative and additive. The multiplicative approach, where beta = gamma/alpha (Jost et al., 2010), assumes that alpha and beta are independent components and could be calculated considering rare (^0^ *D*), typical (^1^ *D*), and dominant (^2^ *D*) species (Jost, 2007; Jost et al., 2010). This approach can be interpreted as the regional beta diversity, as only one value of beta diversity is given for *N* sites. Regional beta diversity of species corresponds to the effective number of completely distinct communities in the region (e.g., the shallow region). For functional diversity, this metric describes the functional differentiation among communities, and for phylogenetic diversity, it expresses the effective number of equally large and completely distinct assemblages, meaning no shared branches between communities (Chao et al., 2014).

For the beta additive approach, we considered the presence/absence of species corresponding to Hill numbers species diversity of order ^0^ *D*. The additive approach produces pairwise beta‐diversity values and was used to partition beta into nestedness and turnover components (Baselga, 2010). In this sense, the multiplicative approach estimated the number of completely distinct communities in the shallow and deep areas, while the additive approach shed light into the mechanisms underlying such patterns. The multiplicative approach was applied to construct the taxonomic, functional, and phylogenetic beta profiles of shallow and deep reefs for rare (^0^ *D*), typical (^1^ *D*), and dominant (^2^ *D*) species, while the additive partitioning of beta species diversity was employed to measure species nestedness and turnover between shallow and deep reefs, as well as within shallow areas and within deep areas. Alpha, gamma, and beta were measured in R using *entropart* (Marcon & Hérault, 2015) for species diversity, the functional diversity R function available in Chiu and Chao (2014), and the package *hillR* (Chao et al., 2014) for phylogenetic diversity estimation.

Finally, we performed a one‐way Wilcoxon test to test whether diversity metrics were higher in deep reefs when compared to their shallow counterparts for rare (^0^ *D*), typical (^1^ *D*), and dominant (^2^ *D*) species, functions, and lineages. Treating depth as a continuous variable in generalized least‐squares regressions produced similar outcomes (Appendix S2). CWM values of shallow and deep reefs were also compared using the one‐way Wilcoxon test. The diversity estimations and Wilcox test were performed in R software (R Core Team, 2018), using the packages *entropart* (Marcon & Hérault, 2015), *cluster* (Becker et al., 1988), *FD* (Becker et al., 1988), and *hillR* (Chao et al., 2014).

## 3 RESULTS

We recorded 3,821 individuals distributed in 85 fish species, 36 families, and 10 orders (Table 2). The 10 most representative families were Labridae (eight species), Haemulidae (7), Labridae: Scarinae (6), Serranidae (6), Carangidae (6), Lutjanidae (5), Pomacentridae (5), Acanthuridae (3), Pomacanthidae (3), and Sciaenidae (3). Ten species represented 70% of individuals: *Haemulon squamipinna* (23%), *Mulloidichthys martinicus* (18%), *Holocentrus adscencionis* (6%), *Kyphosus incisor* (6%), *Acanthurus chirurgus* (4%), *Carangoides bartolomaei* (3%), *Haemulon aurolineatum* (3%), *Lutjanus alexandrei* (3%), *Cephalopholis fulva* (2%), and *Pareques acuminatus* (2%). In terms of vertical distribution, 41 species occurred in both shallow and deep areas, while 15 species occurred only in shallow reefs and 29 only in deep reefs (Table 2), indicating that depth‐generalist species do not dominate the metacommunity. Seven species were listed in the IUCN Red List (Table 2), including *Scarus trispinosus* (endangered) and other three Scarinae species (vulnerable), *Ginglymostoma cirratum* (vulnerable), *Elacatinus figaro* (vulnerable), and *Mycteroperca bonaci* (vulnerable). No nonindigenous or invasive species were recorded.

**TABLE 2:** Fish species recorded in the 22 shallow and deep reefs of Northeast Brazil. Numbers in shallow and deep categories represent species abundance. The last column identifies threatened species according to IUCN’s (the International Union for Conservation of Nature) red list (available at https://www.iucnredlist.org)

Family	Species	Shallow	Deep	IUCN
Ginglymostomatidae	*Ginglymostoma cirratum* (Bonnaterre 1788)		5	VU
Dasyatidae	*Hypanus berthalutzae* (Petean, Naylor & Lima 2020)		2	
	*Hypanus* sp		1	
Muraenidae	*Gymnothorax funebris* (Ranzani 1839)	1	5	
	*Muraena pavonina* (Richardson 1845)	2	1	
Ophichthidae	*Myrichthys ocellatus* (Lesueur 1825)	1	1	
Fistulariidae	*Fistularia tabacaria* (Linnaeus 1758)	1		
Scorpaenidae	*Scorpaena plumieri* (Bloch 1789)		1	
Holocentridae	*Holocentrus adscensionis* (Osbeck 1765)	22	211	
	*Myripristis jacobus* (Cuvier 1829)		64	
Serranidae	*Alphestes afer* (Bloch 1793)	3	2	
	*Cephalopholis fulva* (Linnaeus 1758)	14	68	
	*Epinephelus adscensionis* (Osbeck 1765)	1	11	
	*Mycteroperca bonaci* (Poey 1860)		2	VU
	*Paranthias furcifer* (Valenciennes 1828)		38	
	*Rypticus saponaceus* (Bloch & Schneider 1801)	1	2	
Malacanthidae	*Malacanthus plumieri* (Bloch 1786)		5	
Echeneidae	*Echeneis naucrates* (Linnaeus 1758)	2		
Carangidae	*Caranx bartholomaei* (Cuvier 1833)		129	
	*Caranx latus* (Agassiz 1831)	3		
	*Caranx lugubris* (Poey 1860)		2	
	*Elagatis bipinnulata* (Quoy & Gaimard 1825)		13	
	*Pseudocaranx dentex* (Bloch & Schneider 1801)		2	
	*Selar crumenophthalmus* (Bloch 1793)		57	
Lutjanidae	*Lutjanus alexandrei* (Moura & Lindeman 2007)	20	90	
	*Lutjanus* cf. *apodus* (Walbaum 1792)	1	3	
	*Lutjanus jocu* (Bloch & Schneider 1801)	4	37	
	*Lutjanus synagris* (Linnaeus 1758)		5	
	*Ocyurus chrysurus* (Bloch 1791)		63	
Haemulidae	*Anisotremus moricandi* (Ranzani 1842)	5		
	*Anisotremus surinamensis* (Bloch 1791)		7	
	*Anisotremus virginicus* (Linnaeus 1758)	15	26	
	*Haemulon aurolineatum* (Cuvier 1830)	46	73	
	*Haemulon parra* (Desmarest 1823)	3	14	
	*Haemulon plumierii* (Lacepède 1801)	6	21	
	*Haemulon squamipinna* (Rocha & Rosa 1999)	337	560	
Sparidae	*Calamus pennatula* (Guichenot 1868)		1	
Sciaenidae	*Equetus lanceolatus* (Linnaeus 1758)		1	
	*Odontoscion dentex* (Cuvier 1830)	3	9	
	*Pareques acuminatus* (Bloch & Schneider 1801)	4	68	
Mullidae	*Mulloidichthys martinicus* (Cuvier 1829)	2	682	
	*Pseudupeneus maculatus* (Bloch 1793)	5	43	
Pempheridae	*Pempheris schomburgkii* (Müller & Troschel 1848)	14		
Chaetodontidae	*Chaetodon ocellatus* (Bloch 1787)		7	
	*Chaetodon striatus* (Linnaeus 1758)	5	5	
Pomacanthidae	*Holacanthus ciliaris* (Linnaeus 1758)	5	13	
	*Holacanthus tricolor* (Bloch 1795)	1	23	
	*Pomacanthus paru* (Bloch 1787)	3	10	
Kyphosidae	*Kyphosus incisor* (Cuvier 1831)		229	
Cirrhitidae	*Amblycirrhitus pinos* (Mowbray 1927)	1	6	
Pomacentridae	*Abudefduf saxatilis* (Linnaeus 1758)	15	47	
	*Chromis multilineata* (Guichenot 1853)	8	27	
	*Stegastes fuscus* (Cuvier 1830)	12		
	*Stegastes pictus* (Castelnau 1855)		25	
	*Stegastes variabilis* (Castelnau 1855)	2		
Sphyraenidae	*Sphyraena barracuda* (Edwards 1771)		16	
Labridae	*Bodianus rufus* (Linnaeus 1758)	7	38	
	*Clepticus brasiliensis* (Heiser, Moura & Robertson 2000)		6	
	*Halichoeres brasiliensis* (Bloch 1791)	5	4	
	*Halichoeres dimidiatus* (Agassiz 1831)	3	21	
	*Halichoeres penrosei* (Starks 1913)	1		
	*Halichoeres poeyi* (Steindachner 1867)	9	14	
	*Thalassoma noronhanum* (Boulenger 1890)	1	47	
	*Xyrichtys martinicensis* (Valenciennes 1840)	5		
Labridae: Scarinae	*Cryptotomus roseus* (Cope 1871)	1		
	*Scarus trispinosus* (Valenciennes 1840)	1	5	EN
	*Scarus zelindae* (Moura, Figueiredo & Sazima 2001)	1	7	VU
	*Sparisoma amplum* (Ranzani 1841)		1	
	*Sparisoma axillare* (Steindachner 1878)	24	20	VU
	*Sparisoma frondosum* (Agassiz 1831)	5	10	VU
Opistognathidae	*Opistognathus* sp		2	
Labrisomidae	*Labrisomus nuchipinnis* (Quoy & Gaimard 1824)	3		
Blenniidae	*Ophioblennius trinitatis* (Miranda Ribeiro 1919)	2		
Gobiidae	*Elacatinus figaro* (Sazima, Moura & Rosa 1997)	6	16	VU
Microdesmidae	*Ptereleotris randalli* (Gasparini, Rocha & Floeter 2001)	2		
Acanthuridae	*Acanthurus bahianus* (Castelnau 1855)	27	16	
	*Acanthurus chirurgus* (Bloch 1787)	42	93	
	*Acanthurus coeruleus* (Bloch & Schneider 1801)	11	40	
Scombridae	*Scomberomorus regalis* (Bloch 1793)		1	
Balistidae	*Balistes vetula* (Linnaeus 1758)	1		
Monacanthidae	*Cantherhines macrocerus* (Hollard 1853)		11	
	*Cantherhines pullus* (Ranzani 1842)		4	
Ostraciidae	*Acanthostracion polygonius* (Poey 1876)	1		
Tetraodontidae	*Canthigaster figueiredoi* (Moura & Castro 2002)		4	
	*Sphoeroides spengleri* (Bl och 1785)	1	1	

Abbreviation: EN, endangered; VU, vulnerable.

### 3.1 Gamma diversity

Gamma diversity of rare, typical, and dominant species was smaller in shallow than deep reefs (^0^ *D* Gamma _Shallow_ = 56 vs. Gamma _Deep_ = 70; ^1^ *D* Gamma _Shallow_ = 20.5 vs. Gamma _Deep_ = 29; ^2^ *D* Gamma _Shallow_ = 12.5 vs. Gamma _Deep_ = 17.7). Gamma functional diversity, expressed as the effective total functional diversity, was higher in deep reefs for ^0^ *D* (Gamma _Shallow_ = 1612; Gamma _Deep_ = 2,610), ^1^ *D* (Gamma _Shallow_ = 142; Gamma _Deep_ = 240), and ^2^ *D* (Gamma _Shallow_ = 43; Gamma _Deep_ = 66). Following the same trend, gamma phylogenetic diversity was also higher for deep reefs in all scenarios (^0^ *D* Gamma _Shallow_ = 3,338 vs. Gamma _Deep_ = 3,503; ^1^ *D* Gamma _Shallow_ = 615 vs. Gamma _Deep_ = 804; ^2^ *D* Gamma _Shallow_ = 398 vs. Gamma _Deep_ = 452). In the three types of diversity measured, gamma was consistently higher in deep reefs compared with their shallow counterparts, which might support the DRRH if species composition of shallow reefs was nested within deep reefs, but that was not case (see Section 3.3).

### 3.2 Alpha diversity

**FIGURE 3:**
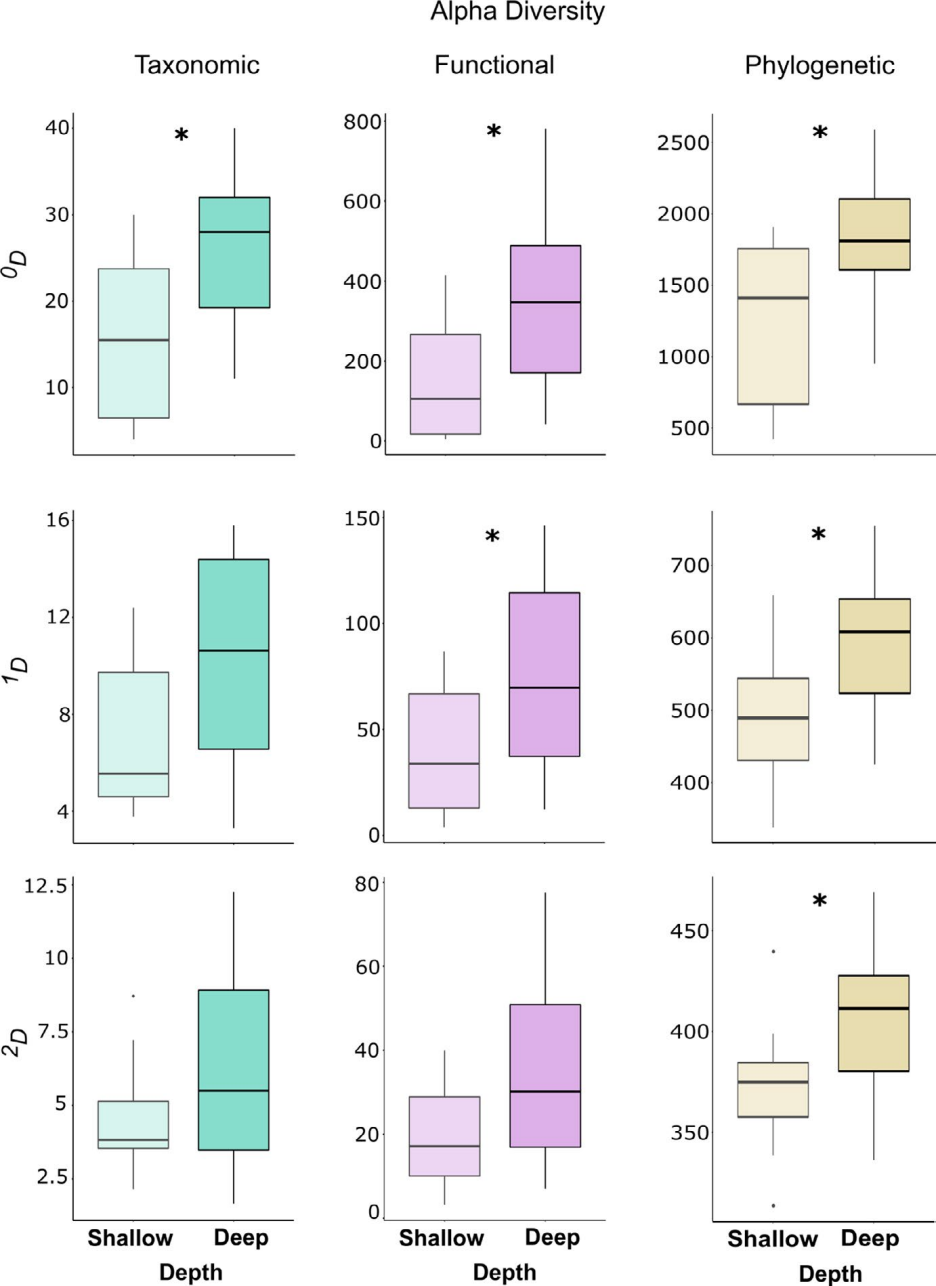
Alpha diversity of rare (^0^ *D*), typical (^1^ *D*), and dominant (^2^ *D*) fish species in shallow (<30 m depth) and deep (>30 m depth) reefs of Northeast Brazil. Asterisk represents significant difference with *p* < 0.05

The mean effective number of rare species was greater in deep than shallow reefs (U = 25, *p* = 0.012), supporting the DRRH for rare species, but did not significantly differ between shallow and deep reefs for typical and dominant species (Figure 3). Alpha functional diversity was also significantly greater for rare (U = 26, *p* = 0.022) and typical species in deep reefs (U = 30, *p* = 0.041), and tended to be greater for dominant species (Figure 3). When we analyzed the data separately by functional trait, differences between shallow and deep reefs were raised for seven trait states (Table S2). Compared with shallow reefs, deep reefs showed significantly more benthic and pelagic species (*U* = 27.5, *p* = 0.023; *U* = 32.5, *p* = 0.049, respectively), larger species (attribute 80 cm <total length >50 cm, *U* = 27.5, *p* = 0.025; attribute total length >80 cm, *U* = 17, *p* = 0.004), and ovoviviparous species (*U* = 28, *p* = 0.012). None of the functional traits were exclusive to the shallow, whereas ovoviviparous was exclusive to deep reefs. Information on all traits is available at supplementary information (Table S2).

Following expectations, alpha phylogenetic diversity was consistently greater in deep reefs than in shallow reefs, considering rare (*U* = 26, *p* = 0.022), typical (*U* = 29, *p* = 0.035), and dominant species (*U* = 31, *p* = 0.047) (Figure 3).

### 3.3 Beta diversity

**FIGURE 4:**
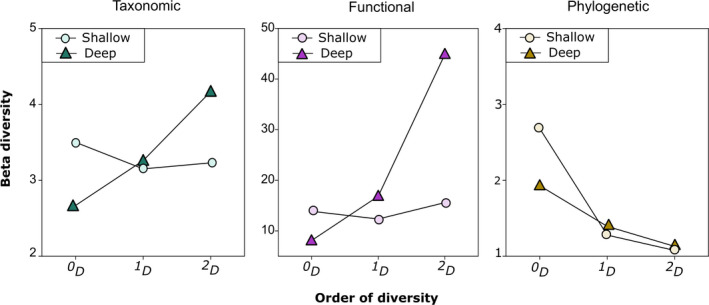
Profiles of taxonomic, functional, and phylogenetic beta diversity of rare (^0^ *D*), typical (^1^ *D*), and dominant (^2^ *D*) fish species in shallow (circle) and deep (triangle) reefs of Northeast Brazil

Regional beta diversity of species and functions showed consistent responses to depth (Figure 4). In the shallow reefs, rare, typical, and dominant species showed similar levels of beta diversity, oscillating around 3.0–3.5 completely distinct communities out of 8 possible (Figure 4). On the other hand, in the deep reefs, regional beta diversity increased dramatically from rare (^0^ *D*) to dominant species (^2^ *D*), reaching 4 completely distinct communities out of 14 possible (Figure 4). These same trends were observed for regional functional beta diversity, indicating that large aggregations—the dominant species—change more at higher depths not only taxonomically but also functionally. Conversely, rare, solitary species tended to be the same at deep waters and perform similar functions when compared to their counterparts in shallow waters.

Regarding the validity of DRRH, the curves of deep reefs in diversity profiles (Figure 4) should be above the curves of shallow reefs to indicate that deep reefs are more heterogeneous than shallow ones, but this was observed only for taxonomic and functional ^2^ *D*. The phylogenetic beta profile also revealed corresponding levels of regional beta diversity for rare and typical species regardless depth. However, beta diversity of dominant species did not increase in deep reefs, contradicting the expectation of phylogenetic homogenization at shallow waters for all orders of diversity.

**FIGURE 5:**
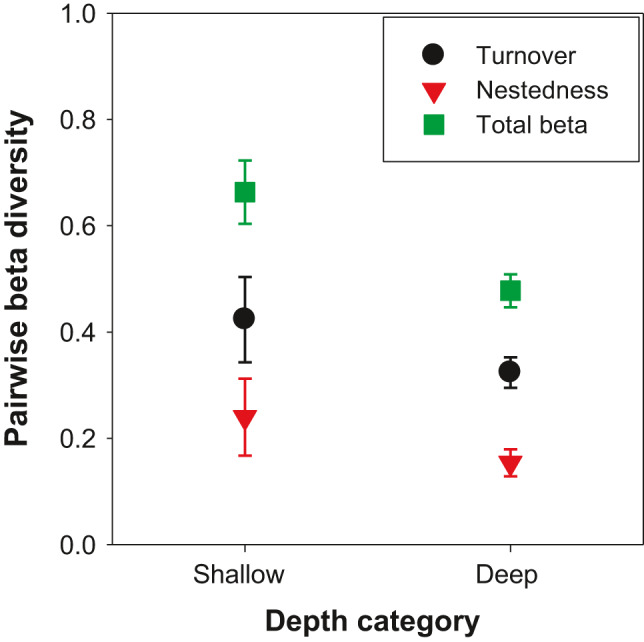
Pairwise beta diversity of shallow and deep reefs decomposed into turnover and nestedness components. Symbols represent mean values between pairs of reefs; the upper and lower error bars indicate 95% confidence interval. Total beta refers to the sum of turnover and nestedness components

When the pairwise beta diversity of species was decomposed into nestedness and turnover components, the spatial variation in species composition was more explained by turnover than nestedness and irrespective to depth (Figure 5). In shallow reefs, turnover accounted on average for 0.42, while nestedness for 0.24 of total beta diversity (Figure 5). Similarly, turnover between deep reefs reached 0.32 on average, while nestedness only 0.15 (Figure 5). Both results were contrary to the DRRH expectation of smaller turnover at shallow waters and indicate that each reef gives its contribution to the gamma diversity.

Taking the beta‐diversity patterns together, it is possible to infer that the larger fish agglomerations at higher depths diverge taxonomically and functionally from a deep reef to another, although they converge phylogenetically toward particular basal clades. Carangidae, for instance, which was almost exclusive from the deep reefs, might be one of those ecologically dominant clades with many species and functions that phylogenetically homogenized the deep reefs.

## 4 DISCUSSION

Our findings indicate that deep reefs may serve as refuge or refugium for some depth‐generalist taxa, functions, and lineages, including some species that use the depth gradient during the ontogenetic migration (Aschenbrenner et al., 2016; Fredou & Ferreira, 2005). However, a representative portion of the fish diversity is exclusive to the shallow reefs or is not evenly distributed across ecologically rare, typical, and dominant groups along the depth gradient, providing limited support to the DRRH. Five findings merit special attention. First, 15 species were exclusive to the shallow reefs, indicating that their local extinction is unlikely to be reverted by immigrants from deep reefs. Second, deep reefs had more rare species than shallow reefs but not more typical and dominant species, suggesting that there are stressors in deeper areas limiting dominance locally. Third, the great contribution of the turnover component to pairwise beta‐diversity patterns and the taxonomic segregation of shallow and deep reefs indicate that the shallow reefs are not a subset from the deep reefs, thus cannot be replenished by deep reefs. Fourth, the functional responses of fish communities to depth resembled the taxonomic responses, revealing similar limitations of shallow and deep reefs to protect regional fish diversity. Fifth, phylogenetic beta diversity suggested that typical and dominant species belong to a few clades irrespective to depth. Jointly, these results highlight that the deep reefs of southwestern Atlantic, as other marginal reefs around the world, have their own dynamics and deserve the same attention that shallow reefs do (Soares et al., 2021).

The number of studies documenting deviations from DRRH expectations and demonstrating the complementary nature of shallow and deep reefs to the shallow‐deep diversity has gradually increased (e.g., Bongaerts & Smith, 2019; Semmler et al., 2017). For instance, Rocha et al. (2018) employed a species composition approach to test the hypothesis with reef fishes of western Atlantic and Pacific and found strong depth specificity for species in the mesophotic zone, indicating that shallow and deep communities were composed by different species rather than a subset of one another. Similar results were found in terms of fish abundance, species richness, trophic groups, and size classes by Pereira et al. (2018) in southwestern Atlantic. In our study region, the DRRH was tested for coral communities with the same analytical approach used here and little support for the hypothesis was observed as well (Morais & Santos, 2018). Nonetheless, we stress that particular species, functions, and lineages may do found refuge or refugium in deep reefs, but not entire fish communities (Soares et al., 2021).

In fact, our findings support the general notion that the shallow reefs diverge taxonomically and functionally from the upper limits of the mesophotic region (30–60 m) (Rocha et al., 2018), which has been also documented in tropical Atlantic (Bejarano et al., 2014; Pinheiro et al., 2016) and Pacific reefs (Coleman et al., 2018; Lindfield et al., 2016). In Gulf of Mexico, for instance, the peak of species turnover takes place at 60 m, which shares only 48% of species with adjacent mesophotic bands at 40–60 and 60–80 m and even less with the shallow area (0–20 m) (Semmler et al., 2017). The authors suggest that this compositional pattern is mostly driven by benthic community composition. This is likely to explain our results as well, but because shallow and deep reefs are several kilometers apart from each other in our study region, it is possible that the structural disconnection between shallow and deep reefs underlies the taxonomic segregation (see also Morais & Santos, 2018).

The phylogenetic dimension of fish diversity may shed light on the evolutionary potential of shallow and deep reefs to adapt to global warming and ocean sprawl (Véron et al., 2019; Winter et al., 2013). Because human stressors are more intense in shallow reefs, and only a small number of disturbance‐tolerant lineages may take advantage of the new conditions in the Anthropocene (Jia et al., 2020; Ribeiro et al., 2016), we expected more phylogenetic homogenization among shallow reefs than among deep reefs. However, this did not happen, suggesting that deep reefs do not count with more evolutionary diversity to couple with ongoing and future changes. Locally, at the alpha scale, the deep reefs do accumulate more lineages than shallow reefs, but this is not enough to face multiple large‐scale disturbances (Albouy et al., 2015). We stress that conservation and management actions of reef environments should incorporate the phylogenetic dimension of fish communities to protect their diversity at any depth. According to our findings, shallow and deep reefs are quite similar in terms of phylogenetic beta‐diversity patterns, with a few ecologically dominant clades homogenizing them throughout the study region.

The increased gamma diversity observed in the deep reefs possibly reflects more suitable conditions for reef fish diversity or represents an important faunal corridor for species associated with deep reef formations across the Atlantic region (Olavo et al., 2011; Soares et al., 2019). Different factors can be related to the increasing gamma diversity from deep to shallow reefs. Most of them are associated with less human pressure in deep areas (Downing et al., 2005; Pereira et al., 2018; Quimbayo et al., 2018; Villéger et al., 2017), but the idea that deep reefs are undisturbed, pristine habitats has been challenged. While the shallow reefs mostly concentrate impacts such as nonregulated tourism, overfishing, and pollution associated with the proximity to the mainland (Downing et al., 2005; Pereira et al., 2018; Quimbayo et al., 2018; Villéger et al., 2017), the deep reefs are usually threatened by invasive species, marine debris, overfishing, and oil/gas exploitation (Soares et al., 2019). Although our study region did not face biological invasion and oil/gas exploitation, fishing gears were observed abandoned in some deep reefs (B.A.S., personal observation). These human stressors in presumably undisturbed deep reefs should have reduced the differences we observe today with the shallow reefs. However, because the shallow reefs of our study region have been facing mass tourism, pollution, and overfishing in the past decades, we attribute their reduced gamma diversity to increased human impacts.

Finally, from the theoretical perspective, the deviation from DRRH expectations suggests that the reef fish metacommunity is not structured by mass effects, although source–sink dynamics and rescue effects should occur for some species. In fact, the unexpected greater contribution of turnover in explaining beta‐diversity patterns at any depth suggests that the metacommunity is mainly structured by species sorting (Leibold et al., 2004). In this model of metacommunity structuring, local environmental conditions are more important than dispersal capacity, allowing species to persist only under suitable conditions. The depth gradient is split into spatial (depth) niches as documented for corals (Morais & Santos, 2018), and the beta diversity becomes high because each local community (i.e., each reef) differs from one another and adds new species to the regional species pool. Because fish distribution along environmental gradients is driven by a product of biogeographic (Floeter et al., 2008; Rocha et al., 2008), historical (Slattery et al., 2011), ecological (Beukers & Jones, 1998), and abiotic factors (Darling et al., 2017), further studies are needed to understand the real role of species sorting across the southwestern Atlantic reefs. However, whatever the driver of diversity patterns, our study highlights that shallow and deep reefs complement each other and must be managed and protected accordingly.

## CONFLICT OF INTEREST

The authors declare that there is no conflict of interest.

## AUTHOR CONTRIBUTION

**Aline P. M. Medeiros:** Conceptualization (lead); Formal analysis (lead); Funding acquisition (lead); Investigation (lead); Methodology (lead); Writing‐original draft (lead); Writing‐review & editing (lead). **Beatrice Ferreira:** Data curation (supporting); Methodology (supporting); Supervision (supporting); Writing‐review & editing (supporting). **Ricardo Betancur:** Formal analysis (supporting); Methodology (supporting); Software (supporting); Supervision (supporting); Writing‐review & editing (supporting). **Fredy Alvarado:** Formal analysis (equal); Methodology (equal); Writing‐review & editing (equal). **Marcelo Soares:** Investigation (equal); Writing‐review & editing (equal). **Braulio A. Santos:** Conceptualization (lead); Formal analysis (lead); Funding acquisition (lead); Investigation (lead); Methodology (lead); Supervision (lead); Writing‐original draft (lead); Writing‐review & editing (lead).

## ETHICAL STATEMENT

Research authorization was obtained via protocols SISBIO # 71158 and SUDEMA # 5192/19.

## Supporting information

Appendix S1‐S2

## Data Availability

Data are available in the Dryad Digital repository at https://datadryad.org/stash/share/plf6k7Mz8oApKifT67o6nUTNwJFZ6mVJLfjw96BpvxE.
