# Supplementary material for: Deep reefs are not refugium for shallow‐water fish communities in the southwestern Atlantic
Source: Ecol Evol. 2021 Mar 18;11(9):4413–27. doi: 10.1002/ece3.7336 (PMC8093723; doi:10.1002/ece3.7336)
Supplement: Supplementary file 1 — Appendix S1‐S2 [file ECE3-11-4413-s001.docx]

Ecology and Evolution – Supplementary material

**Deep reefs are not refugium for shallow-water fish communities in the southwestern Atlantic**

Aline P. M. Medeiros^1^, Beatrice P. Ferreira^2^, Fredy Alvarado^3^, Ricardo Betancur-R^4,5^, Marcelo O. Soares^6,7,8^, Bráulio A. Santos^9^*

^1^ Programa de Pós-Graduação em Ciências Biológicas, Universidade Federal da Paraíba, Cidade Universitária, Castelo Branco, 58051-900, João Pessoa, PB, Brazil

^2^ Departamento de Oceanografia, Centro de Tecnologia, Universidade Federal de Pernambuco, Cidade Universitária, 50670-901, Recife, PE, Brazil

^3^ Departamento de Agricultura, Centro de Ciências Humanas, Sociais e Agrárias, Universidade Federal da Paraíba, Campus Universitário III, R. João Pessoa, S/N, 58220-000, Bananeiras, PB, Brazil

^4^ Department of Biology, The University of Oklahoma, 730 Van Vleet Oval, Room 314, Norman, OK 73019, USA

^5^ Department of Vertebrate Zoology, National Museum of Natural History, Smithsonian Institution, Washington DC 20560, USA

^6^ Instituto de Ciências do Mar-LABOMAR, Universidade Federal do Ceará, Av. da Abolição, 3207, Meireles, 60165-081, Fortaleza, CE, Brazil

^7^ Institut de Ciència i Tecnologia Ambientals (ICTA), Universitat Autònoma de Barcelona (UAB), Carrer de les Columnes, Edifici Z, Barcelona, Spain

^8^ Dipartimento di Scienze e Tecnologie Biologiche e Ambientali (DISTEBA), Università del Salento, Lecce, Italy

^9^ Departamento de Sistemática e Ecologia, Centro de Ciências Exatas e da Natureza, Universidade Federal da Paraíba, Cidade Universitária, Castelo Branco, 58051-900, João Pessoa, PB, Brazil.

*****Corresponding author: braulio@dse.ufpb.br

**Appendix S1.** Complementary species composition analyses.

We carried out species preliminary species composition analyses according to depth categories and geographic distance. Prior to these analyses, data were log(x + 1) transformed to reduce discrepancies caused by rare and abundant species and then used in the construction of similarity matrices (Bray–Curtis index) in PRIMER 6.0 Software (Clarke & Warwick, 2001). To assess the relationship between species composition and depth categories, we performed an ANOSIM test and a non-metric dimensional scaling (NMDS). Mantel test was applied to assess if geographic distance and depth difference between reefs were correlated with species similarity. While species composition was not correlated with geographic distance between reefs (Rho= 0.098; p> 0.05), depth presented a significant correlation with species similarity (Rho= 0.542; p= 0.001), indicating that reefs at similar depth tended to be more taxonomically similar than reefs at different depths. This effect of depth on species composition was visible in the NMDS and supported by the ANOSIM (Global_R_= 0.439, p= 0.001). The functional distance between reefs was not related to difference in depth (Rho= 0.026; p> 0.05) or geographical distance (Rho= -0.154; p> 0.05).

Non-metric multidimensional scaling (NMDS) applied to the reef fish communities of shallow (< 30 m) and deep (> 30 m) reefs in Northeast Brazil.

**Appendix S2.** Generalized least square modelling applied to test the effect of depth on diversity metrics.

Besides testing whether species, functional and phylogenetic alpha diversity were related to depth categories (shallow vs. deep), we performed generalized least square (GLS) analyses treating depth as a continuous variable. Depth and diversity values were log-transformed (natural log) prior to the analyses. The results we obtained using Wilcox tests were similar to the GLS outcomes, with alpha diversity being positively correlated to the increase in depth for ^0^*D* in species diversity; ^0^*D,* ^1^*D* and ^2^*D* in functional diversity; ^0^*D*, ^1^*D* and ^2^*D* in phylogenetic diversity.

Results of the GLSs applied to taxonomic, functional and phylogenetic alpha diversity according to reef depth. The level of significance is indicated as follows: *< 0.05; **< 0.01; NS – not significant.

| Diversity type | *q* order | R-square | *P* | Significance |
| --- | --- | --- | --- | --- |
| Taxonomic | ^0^*D* | 0.2668 | 0.01385 | * |
| Taxonomic | ^1^*D* | 0.06745 | 0.243121 | NS |
| Taxonomic | ^2^*D* | 0.01669 | 0.5667 | NS |
| Functional | ^0^*D* | 0.2829 | 0.0108 | * |
| Functional | ^1^*D* | 0.2431 | 0.01971 | * |
| Functional | ^2^*D* | 0.1875 | 0.04410 | * |
| Phylogenetic | ^0^*D* | 0.3602 | 0.00315 | ** |
| Phylogenetic | ^1^*D* | 0.2452 | 0.0191 | * |
| Phylogenetic | ^2^*D* | 0.1855 | 0.0454 | * |

**Table S1.** Species included in the time-calibrated tree, estimated using maximum likelihood and backbone constraint analyses. The source (B17) corresponds to species already placed in the backbone tree (Betancur-R et al., 2017); the remaining species (GenBank) were placed based on *cytochrome oxidase subunit I* (COI) and *cytochrome b* (Cytb) sequences obtained from NCBI. NCBI accession numbers are provided.

| **Species (as in GenBank)** | **Species** | **Source** | **COI** | **Cytb** | |
| --- | --- | --- | --- | --- | --- |
| *Abudefduf saxatilis* | *Abudefduf saxatilis* | B17 |  |  | |
| *Acanthostracion polygonius* | *Acanthostracion polygonius* | GenBank | JQ861011.1 | JQ861154.1 | |
| *Acanthurus bahianus* | *Acanthurus bahianus* | B17 |  |  | |
| *Acanthurus chirurgus* | *Acanthurus chirurgus* | GenBank | JQ842356.1 | KC623696.1 | |
| *Acanthurus coeruleus* | *Acanthurus coeruleus* | GenBank | JQ842776.1 | KC623697.1 | |
| *Alphestes afer* | *Alphestes afer* | GenBank | JQ840759.1 | AY313996.1 | |
| *Amblycirrhitus pinos* | *Amblycirrhitus pinos* | B17 |  |  | |
| *Anisotremus moricandi* | *Anisotremus moricandi* | GenBank | JQ741142.1 | EU694316.1 | |
| *Anisotremus surinamensis* | *Anisotremus surinamensis* | B17 |  |  | |
| *Anisotremus virginicus* | *Anisotremus virginicus* | B17 |  |  | |
| *Balistes vetula* | *Balistes vetula* | B17 |  |  | |
| *Bodianus rufus* | *Bodianus rufus* | GenBank | JQ839717.1 |  | |
| *Calamus pennatula* | *Calamus pennatula* | GenBank | KY402425.1 | |  |
| *Cantherhines macrocerus* | *Cantherhines macrocerus* | GenBank | JQ842801.1 |  | |
| *Cantherhines pullus* | *Cantherhines pullus* | GenBank | MF041486.1 | KF025770.1 | |
| *Canthigaster figueiredoi* | *Canthigaster figueiredoi* | GenBank | JQ681776.1 | JQ681871.1 | |
| *Carangoides bartholomaei* | *Carangoides bartholomaei* | GenBank | JQ841092.1 | AY050728.1 | |
| *Caranx latus* | *Caranx latus* | GenBank | JQ841100.1 | AY050724.1 | |
| *Caranx lugubris* | *Caranx lugubris* | GenBank | MK566835.1 | |  |
| *Cephalopholis fulva* | *Cephalopholis fulva* | B17 |  |  | |
| *Chaetodon ocellatus* | *Chaetodon ocellatus* | B17 |  |  | |
| *Chaetodon striatus* | *Chaetodon striatus* | B17 |  |  | |
| *Chromis multilineata* | *Chromis multilineata* | GenBank | JQ842056.1 | EU431997.1 | |
| *Cryptotomus roseus* | *Cryptotomus roseus* | B17 |  |  | |
| *Echeneis naucrates* | *Echeneis naucrates* | B17 |  |  | |
| *Elacatinus figaro* | *Elacatinus figaro* | GenBank | KM987237.1 | AY846438.1 | |
| *Elagatis bipinnulata* | *Elagatis bipinnulata* | B17 |  |  | |
| *Epinephelus adscensionis* | *Epinephelus adscensionis* | GenBank | FJ583396.1 |  | |
| *Equetus lanceolatus* | *Equetus lanceolatus* | GenBank | KP722721.1 | KP722629.1 | |
| *Fistularia tabacaria* | *Fistularia tabacaria* | B17 |  |  | |
| *Gymnothorax funebris* | *Gymnothorax funebris* | GenBank | JQ842871.1 |  | |
| *Haemulon aurolineatum* | *Haemulon aurolineatum* | B17 |  |  | |
| *Haemulon parra* | *Haemulon parra* | GenBank | JQ841906.1 | EU697512.1 | |
| *Haemulon plumieri* | *Haemulon plumieri* | B17 |  |  | |
| *Haemulon squamipinna* | *Haemulon squamipinna* | GenBank | EU697544.1 | EU697517.1 | |
| *Halichoeres brasiliensis* | *Halichoeres brasiliensis* | GenBank |  | AY823576.1 | |
| *Halichoeres cyanocephalus* | *Halichoeres dimidiatus* | GenBank | JQ841215.1 | AY591376.1 | |
| *Halichoeres maculipinna* | *Halichoeres penrosei* | GenBank | JQ840106.1 | AY591354.1 | |
| *Halichoeres poeyi* | *Halichoeres poeyi* | GenBank | JQ841595.1 | AY823578.1 | |
| *Holacanthus ciliaris* | *Holacanthus ciliaris* | B17 |  |  | |
| *Holacanthus tricolor* | *Holacanthus tricolor* | B17 |  |  | |
| *Holocentrus adscensionis* | *Holocentrus adscensionis* | GenBank | JQ842166.1 | KX961691.1 | |
| *Kyphosus incisor* | *Kyphosus incisor* | B17 |  |  | |
| *Labrisomus nuchipinnis* | *Labrisomus nuchipinnis* | GenBank | GU225343.1 | |  |
| *Lutjanus alexandrei* | *Lutjanus alexandrei* | GenBank | MG575213.1 | |  |
| *Lutjanus apodus* | *Lutjanus* cf. *apodus* | GenBank | JQ842558.1 | U26957.1 | |
| *Lutjanus jocu* | *Lutjanus jocu* | GenBank | KF633372.1 | HQ162442.1 | |
| *Lutjanus synagris* | *Lutjanus synagris* | GenBank | KF633283.1 | HQ162427.1 | |
| *Malacanthus plumieri* | *Malacanthus plumieri* | B17 |  |  | |
| *Mulloidichthys martinicus* | *Mulloidichthys martinicus* | B17 |  |  | |
| *Mycteroperca bonaci* | *Mycteroperca bonaci* | B17 |  |  | |
| *Myrichthys ocellatus* | *Myrichthys ocellatus* | GenBank | JQ842250.1 |  | |
| *Myripristis jacobus* | *Myripristis jacobus* | GenBank | JQ842252.1 | DQ379998.1 | |
| *Ocyurus chrysurus* | *Ocyurus chrysurus* | B17 |  |  | |
| *Odontoscion dentex* | *Odontoscion dentex* | B17 |  |  | |
| *Ophioblennius trinitatis* | *Ophioblennius trinitatis* | GenBank |  | MF990196.1 | |
| *Paranthias furcifer* | *Paranthias furcifer* | GenBank | JQ365485.1 |  | |
| *Pareques acuminatus* | *Pareques acuminatus* | B17 |  |  | |
| *Pempheris schomburgki* | *Pempheris schomburgki* | B17 |  |  | |
| *Pomacanthus paru* | *Pomacanthus paru* | GenBank | JQ840654.1 |  | |
| *Pseudocaranx dentex* | *Pseudocaranx dentex* | GenBank | EF609442.1 | DQ197985.1 | |
| *Pseudupeneus maculatus* | *Pseudupeneus maculatus* | B17 |  |  | |
| *Rypticus saponaceus* | *Rypticus saponaceus* | B17 |  |  | |
| *Scarus trispinosus* | *Scarus trispinosus* | GenBank | MF999162.1 | |  |
| *Scomberomorus regalis* | *Scomberomorus regalis* | B17 |  |  | |
| *Scorpaena plumieri* | *Scorpaena plumieri* | GenBank | JQ365552.1 |  | |
| *Selar crumenophthalmus* | *Selar crumenophthalmus* | B17 |  |  | |
| *Sparisoma amplum* | *Sparisoma amplum* | GenBank |  | DQ457024.1 | |
| *Sparisoma axillare* | *Sparisoma axillare* | GenBank |  | DQ457034.1 | |
| *Sparisoma frondosum* | *Sparisoma frondosum* | GenBank |  | DQ457032.1 | |
| *Sphoeroides spengleri* | *Sphoeroides spengleri* | GenBank | JQ681816.1 | JQ681909.1 | |
| *Sphyraena barracuda* | *Sphyraena barracuda* | B17 |  |  | |
| *Stegastes fuscus* | *Stegastes fuscus* | B17 |  |  | |
| *Stegastes pictus* | *Stegastes pictus* | GenBank | KM077183.1 | KM077201.1 | |
| *Stegastes variabilis* | *Stegastes variabilis* | GenBank | JQ841972.1 | KM077204.1 | |
| *Thalassoma noronhanum* | *Thalassoma noronhanum* | GenBank | JQ839625.1 | AY328876.1 | |
| *Xyrichtys martinicensis* | *Xyrichtys martinicensis* | GenBank | JQ839657.1 | U92005.1 | |

**Table S2.** Community trait weighted mean (CWM) for each state of the six functional traits evaluated in Northeast Brazil. We compared all attributes between shallow and deep reefs using one-way Wilcox-tests. Seven trait states were significantly different between depth categories. Level of significance (*p*): *< 0.05.

|  |  | **CWM average** | |  |  | |
| --- | --- | --- | --- | --- | --- | --- |
| **Trait** | **State** | **Shallow**  **(min, max)** | **Deep**  **(min, max)** | **U** | ***p*** | |
| Water column position | Benthic | 0.05 (0, 0.14) | 0.2 (0.03, 0.8) | 27.5 | 0.028 | * |
|  | Benthopelagic | 0.9 (0.86, 1.0) | 0.7 (0.15, 0.96) | 103 | 0.999 |  |
|  | Pelagic | 0.01 (0, 0.03) | 0.12 (0, 0.62) | 32.5 | 0.049 | * |
| Habitat use | Specialist | 0.17 (0, 0.7) | 0.08 (0, 0.58) | 63 | 0.71 |  |
|  | Intermediate | 0.18 (0.08, 0.3) | 0.31 (0.01, 0.76) | 43.5 | 0.21 |  |
|  | Generalist | 0.65 (0.18, 0.93) | 0.61 (0.24, 0.91) | 61 | 0.65 |  |
| Body size | 0 - 7 cm | 0.01 (0, 0.09) | 0.005 (0, 0.03) | 45.5 | 0.19 |  |
|  | 7.1 - 15 cm | 0.25 (0, 0.73) | 0.14 (0, 0.57) | 56 | 0.76 |  |
|  | 15.1 - 30 cm | 0.48 (0.11, 0.66) | 0.27 (0.01, 0.61) | 83 | 0.97 |  |
|  | 30.1 - 50 cm | 0.23 (0, 0.51) | 0.41 (0.08, 0.87) | 36 | 0.092 |  |
|  | 50.1 - 80 cm | 0.006 (0, 0.028) | 0.08 (0, 0.53) | 27.5 | 0.025 | * |
|  | > 80 cm | 0.02 (0, 0.11) | 0.1 (0, 0.41) | 17 | 0.004 | * |
| Mobility | High mobility | 0.007 (0, 0.036) | 0.06 (0, 0.26) | 33.5 | 0.057 |  |
|  | Roving | 0.89 (0.66, 1) | 0.89 (0.71, 1) | 58.5 | 0.58 |  |
|  | Sedentary | 0.11 (0, 0.33) | 0.05 (0, 0.21) | 72.5 | 0.27 |  |
| Trophic category | Herbivore | 0.24 (0, 0.61) | 0.19 (0, 0.5) | 67 | 0.78 |  |
|  | Macro carnivore | 0.096 (0, 0.011) | 0.19 (0.05, 0.49) | 29 | 0.035 | * |
|  | Macro invertivore | 0.56 (0.16, 0.76) | 0.47 (0.09, 0.87) | 64.5 | 0.73 |  |
|  | Omnivore | 0.01 (0, 0.067) | 0.03 (0, 0.136) | 42 | 0.17 |  |
|  | Planktivore | 0.05 (0, 0.33) | 0.07 (0, 0.21) | 31 | 0.044 | * |
|  | Small invertivore | 0.03 (0, 0.07) | 0.04 (0, 0.14) | 45.5 | 0.25 |  |
| Larval dispersion | Balistidae type | 0.014 (0, 0.06) | 0.015 (0, 0.07) | 50 | 0.34 |  |
|  | Brooding | 0 (0, 0) | 0.0004 (0, 0.006) | 52 | 0.25 |  |
|  | Demersal eggs | 0.11 (0, 0.3) | 0.075 (0, 0.24) | 64 | 0.72 |  |
|  | Pelagic eggs | 0.88 (0.66, 1) | 0.9 (0.71, 1) | 48.5 | 0.32 |  |
|  | Ovoviviparous | 0 (0, 0) | 0.0046 (0, 0.03) | 28 | 0.011 | * |
